# Drip Irrigation and Fertilization Strategies Shape Soil Bacterial Community and Nutrient Utilization in Summer Peanut

**DOI:** 10.3390/plants15192949

**Published:** 2026-09-27

**Authors:** Juan Liu, Yilong Luo, Jingjing Zhang, Haijiao Liu, Yanan Cui, Kun Hu, Nianyuan Jiao, Yangrui Ou, Risheng Xu, Tengfei Ma, Yafei Chen, Bing Liu, Mengwei Zhang, Xinyou Zhang

**Affiliations:** 1 Peanut Institute of Henan Academy of Agricultural Sciences/Postgraduate Training Base of Zhengzhou University, Zhengzhou/Huanghuaihai Key Laboratory of Oil Crops, Ministry of Agriculture and Rural Areas/Henan Key Laboratory of Genetic Improvement of Oil Crops, Zhengzhou 450002, China; liujuanviolet@163.com (J.L.); lyl2388036843@163.com (Y.L.); zhangjingjing0827@163.com (J.Z.); ksmd21lxl@163.com (Y.C.); hukun2017@126.com (K.H.); shengrixu@126.com (R.X.); mtf0716@163.com (T.M.); hnpp2010@163.com (Y.C.); q58372849htjik@163.com (B.L.); 2 School of Agricultural and Biomanufacturing, Zhengzhou University, Zhengzhou 450001, China; m19839687671@163.com; 3 School of Agriculture, Henan University of Science and Technology, Luoyang 471023, China; nianyuanjiao@haust.edu.cn; 4 School of Life Sciences, Zhengzhou University, Zhengzhou 450001, China; oyr2001163@163.com; 5 National Innovation Centre for Bio-Breeding Industry, Zhengzhou 450002, China; 6 The Shennong Laboratory, Zhengzhou 450002, China

**Keywords:** summer peanut, fertilization strategy, drip irrigation, soil bacterial community, enzyme activity, nutrient uptake and utilization

## Abstract

Optimizing fertilization strategies is key to increasing peanut yields and maintaining soil health, but it is still unclear how the proportion of basal fertilizer (applied before sowing) and topdressing (applied through drip irrigation during growth) systematically affects the “soil microbe plant” system under integrated drip irrigation and water–fertilizer management. This study set up four fertilization strategies—no fertilizer (F1), full basal fertilizer (F2: N–P_2_O_5_–K_2_O = 343.9–123.0–120.0 kg·hm^−2^ applied at sowing), drip irrigation with full water-soluble fertilizer (F3: total NPK input same as F2, applied via drip irrigation at flowering–pegging, pod-setting, and pod-filling stages in a 6:2:2 ratio), and 50% basal fertilizer + 50% water-soluble fertilizer (F4: basal applied at sowing, water-soluble applied via drip irrigation at the three growth stages in a 2:4:4 ratio)—to explore their effects on the soil nutrient availability, enzyme activity, bacterial community, and plant nutrient uptake throughout the entire growth stage of peanuts. The results showed that drip irrigation and topdressing strategies significantly improved soil-available nutrients and enzyme activity in the later stages of growth, with the F4 treatment showing the most significant advantage. This treatment not only significantly improved bacterial diversity and community functional redundancy and maintained a stable presence of multifunctional beneficial bacterial genera but also significantly increased nitrogen, phosphorus, and potassium uptake and fertilizer use efficiency. Partial least-squares structural equation modeling (PLS-SEM) reveals that fertilization strategies mainly indirectly drive yield increase by regulating microbial communities and enzyme activity. In summary, under the condition of equal nutrient input, the strategy of evenly matching nutrient supply with peanut demand was achieved, which not only ensured high yield and efficiency but also enhanced soil microecological stability. It is the optimal strategy for integrating water and fertilizer into the drip irrigation of summer peanuts under the conditions of this experiment.

## 1. Introduction

Peanut is an important oil and cash crop in China. It has high oil and edible value and plays an important role in ensuring the safety of national grain and oil [1]. However, the long-standing problem of extensive nutrient management in peanut production has become a key bottleneck restricting its yield potential and soil health. As a leguminous crop, peanut can form symbiotic nodules with rhizobia to fix atmospheric nitrogen, thereby partially meeting its nitrogen demand. However, excessive nitrogen fertilizer application is known to suppress nodulation and nitrogen fixation activity, potentially reducing the contribution of biological nitrogen fixation to peanut growth [2,3,4,5].

Peanut’s nutrient demand exhibits a dynamic pattern: low at the seedling stage, increasing at the flowering–pegging stage, and peaking at the pod-setting stage. The peak of nutrient requirement is concentrated in the middle and late growth stages; however, traditional fertilization mostly uses one-time basal fertilization before sowing, which easily leads to the “dislocation of supply and demand” problem of excessive nutrients in the early stage and easy leaching in the late stage, as well as nutrient depletion and premature aging, which not only greatly reduces the fertilizer utilization efficiency but also increases the risk of soil degradation [6]. By accurately transporting soluble fertilizer and irrigation water to the crop root zone, drip irrigation water and fertilizer integration technology can effectively realize the coupling of the water and fertilizer supply on demand, which can not only avoid the fixed leaching loss of nutrients in the early stage but also ensure the peak demand of nutrients in the middle and late stages. It has been demonstrated that compared with traditional flood irrigation, drip irrigation can save 25% water and significantly increase production [7,8]. At present, research on the effect of drip irrigation and fertilization on peanut yields and nutrient uptake has been accumulated [9,10,11], but the internal soil driving mechanism of yield difference under different drip irrigation and fertilization strategies is not clear, which restricts the optimization of a precise management scheme of water and fertilizer integration. On peanut, drip fertilization has been proven to significantly improve yield, nutrient uptake and fertilizer agronomic efficiency.

The uptake of plant nutrients and fertilizer utilization efficiency are the direct basis for evaluating the advantages and disadvantages of fertilization strategies, and they are also the key areas of concern in current drip irrigation peanut research. The peak stage of peanut nutrient demand is concentrated from the pod-setting stage to the full fruiting stage [12]. Jain et al. [13] found that the nutrient uptake and yield of peanuts under drip irrigation fertilization were significantly higher than those under conventional fertilization, and the best effect was achieved through staged drip irrigation fertilization. However, the above studies mostly focus on the surface description of yield and uptake, and there is still a lack of sufficient evidence on the underlying biological driving mechanism demonstrating whether the yield gain is due to the systematic improvement in the soil fertilization capacity. Soil enzymes, as one of the key factors affecting soil nutrient conversion, play an important role in nutrient conversion, organic matter decomposition, and metabolic activity [14,15]. Research has shown that optimizing fertilization can significantly increase the available phosphorus and potassium content, as well as urease and alkaline phosphatase activities, in continuous peanut soil, promoting dry matter accumulation and yield improvement [16]. However, changes in enzyme activity are not isolated events, and their underlying causes often point to the structural and functional responses of soil microbial communities. Soil microorganisms are sensitive to environmental changes and to adjusting fertilization properties and enzyme activity [17,18,19,20]. The impact of fertilization strategies on peanut rhizobia is particularly noteworthy. The symbiotic nitrogen fixation system between peanuts and rhizobia contributes a considerable proportion of the nitrogen required by plants, and fertilization measures significantly regulate the efficiency of this symbiotic relationship [21]. In addition, Pseudomonas and Bacillus are the most common non-rhizobial endophytic genera in peanut nodules. Although they cannot independently induce nodulation, they can indirectly promote peanut growth and nodulation through secretion of plant hormones, dissolution of insoluble phosphorus, and production of iron carriers [22]. Previous studies have shown that fertilization measures can significantly affect the bacterial diversity and community composition of peanut continuous cropping soil [23], and the integrated drip irrigation treatment of water and fertilizer can affect crop yields by regulating the abundance of beneficial bacteria [24]. However, current research on microbial response under drip irrigation fertilization is either limited to a single time point or only focuses on final yields. A comprehensive report on the complete temporal coupling system of “fertilization dynamic strategy—soil nutrient evolution during growth stage—enzyme activity fluctuations—bacterial community succession—plant uptake and utilization” is still lacking.

In summary, current research on the effects of drip irrigation fertilization on peanuts mainly focuses on surface level descriptions of yields and fertilizer utilization efficiency, while comprehensive studies that consider “fertilization dynamic strategies—soil nutrient evolution during growth stage, enzyme activity fluctuations, bacterial community succession, plant uptake and utilization” as a complete time-coupled system are still lacking. Partial least-squares structural equation modeling (PLS-SEM) is a statistical method used to test complex causal relationships between multiple variables [25], and it was employed to decompose the direct, indirect, and total effects of fertilization strategies on yields through the “microbial, community, enzyme activity, nutrient availability, plant uptake” cascade pathway in this experiment. The aim is to elucidate the mechanisms of drip irrigation fertilization strategies driving yield formation through the “soil microbial enzyme nutrient plant” cascade pathway, and to provide a scientific basis for the precise management of water and fertilizer integration in summer peanuts.

## 2. Results

### 2.1. Effects of Fertilization Strategies on Available Nutrients in Peanut Soil

As shown in Figure 1, the content of alkali-hydrolyzed nitrogen, available phosphorus, and available potassium in each treated soil reaches its peak during the flowering–pegging stage and then gradually decreases. During the flowering–pegging stage, F3 exhibited the highest level of available nutrients. Starting from the pod-setting stage, the nutritional contents of F3 and F4 were significantly higher than that of F2: alkaline-hydrolyzed nitrogen was 6.82% and 3.96% higher, respectively; the available phosphorus levels were 17.04% and 15.63% higher, respectively; the available potassium levels were 30.98% and 29.80% higher, respectively. It is worth noting that F4 exhibited the greatest advantage during the pod-setting and maturity stages, especially in terms of available phosphorus and potassium. Overall, in the later stage of growth, the soil-available nutrient levels of topdressing drip irrigation (F3 and F4) were significantly higher than those of disposable base fertilizer (F2).

### 2.2. Effect of Fertilization Strategy on Soil Enzyme Activity of Peanut

As shown in Figure 2,from the flowering–pegging stage to the maturity stage, the activities of soil urease (Figure 2a) and sucrase (Figure 2b) gradually decrease, while the activity of alkaline phosphatase (Figure 2c) first increases and then decreases. During the flowering–pegging stage, the urease and sucrase activities of F3 and F4 were significantly higher than those of F2: the urease activities were 6.85% and 2.74% higher, respectively; the activities of sucrase were 14.38% and 8.31% higher, respectively. From the pod-setting stage to the pod-filling stage, the advantages of F3 and F4 are more obvious than those of F2, with F4 showing the highest activity in the later stage. In the maturity stage, the enzyme activity in all treatments reached the lowest level, but that of F3 and F4 was still significantly higher than that of F2. For alkaline phosphatase, F3 showed the highest activity during the flowering–pegging stage, and there was no significant difference between F3 and F4. There was also no significant difference between fertilization treatments (F2–F4) from the pod-setting stage to the pod-filling stage. Overall, in terms of maintaining high soil enzyme activity during the later stages of growth, topdressing and drip irrigation (F3, F4) are more effective than one-time basal fertilizers.

For alkaline phosphatase activity(Figure 2c), the F3 treatment was the highest at the flowering–pegging stage, which was significantly increased by 20.97% compared with the F2 treatment, but there was no significant difference between the F4 treatment and F3 treatment. There was no significant difference between fertilization treatments (F2–F4) from the pod-setting stage to the pod-filling stage. The above results showed that drip irrigation and topdressing (F3, F4) were more conducive to maintaining higher soil enzyme activity at the late growth stage of peanut than one-time basal fertilization (F2).

### 2.3. Effects of Fertilization Strategies on Soil Microbial Community Characteristics

#### 2.3.1. Effects of Fertilization Strategies on Microbial Diversity in Peanut Soil

Figure 3 shows the Chao index and Shannon index for peanut soil under different fertilization conditions. The Chao index and Shannon index of the F4 treatment were the highest at the pod-setting stage and pod-filling stage, but only the Chao index (Figure 3a) of the F4 treatment was significantly higher than those of the F2 and F3 treatments: 9.13% and 9.34% higher, respectively. There was no significant difference between the F3 treatment and F2 treatment.

NMDS analysis is shown in Figure 4. The sample points of each treatment are obviously clustered according to the fertilization treatment, and the separation trend between groups is clear. As shown in the NMDS ranking diagram of the pod setting stage in Figure 4a, there were significant differences in the bacterial community structures among the four fertilization treatments. In the ordination chart, the F1 treatment is significantly separated from the F2–F4 fertilization treatments along the nmds1 axis, indicating that fertilization significantly affected the separation of the bacterial community structure at the pod-setting stage.

As shown in Figure 4b, at the pod-filling stage, the community differentiation among the treatments was further intensified. Compared with the pod-setting stage, the R value of the ANOSIM increased from 0.623 to 0.883, an increase of 41.7%, indicating that the differential effect of the fertilization strategy on bacterial community continued to accumulate and amplify with the advance of the growth stage. The intragroup dispersion of the F4 treatment was the smallest in the pod-filling stage, and the intragroup dispersion of the F3 treatment was significantly greater than that of F4 in the pod-filling stage. The distance between the F1 treatment and F2–F4 treatments in the pod-filling stage was larger than that in the pod-setting stage. The F3 and F4 treatments still had a certain degree of overlap at the pod-setting stage and showed obvious separation at the pod-filling stage.

#### 2.3.2. Effects of Fertilization Strategies on Microbial Community Structure in Peanut Soil

As shown in Figure 5, the main dominant bacteria in the soil bacterial community structure of each treatment at the pod-setting stage and pod-filling stage are Pseudomonadota, Acidobacteriota, Bacillota and Actinomycetota. The significance among treatments is shown in Figure S1.

Figure 5a shows the top 15 bacterial phyla with relative abundance during the pod setting stage.The relative abundance of Pseudomonadota was the highest in the F4 treatment at the pod-setting stage, but there was no significant difference between F4 and the other treatments. The relative abundance of Acidobacteriota in the F3 treatment was the highest, and there was no significant difference with the F1 treatment. The relative abundances in the F1 and F3 treatments were significantly higher than those in the F2 and F4 treatments: 2.93%, 2.46% and 3.58%, 3.11% higher, respectively. The relative abundance of Bacillota in the F2 treatment was the highest (19.51%) and was significantly higher than those in the other treatments. The relative abundances of Bacillota in the F3 and F4 treatments had no significant difference but were significantly higher than that in the F1 treatment. There was no significant difference in the relative abundances of Actinomycetota among the treatments, with the highest value of 9.70% in F3 and the lowest value of 8.43% in F4.

Figure 5b shows the top 15 bacterial phyla with relative abundance during the pod filling stage.The relative abundances of Pseudomonadota in the F2, F3 and F4 treatments at the pod-filling stage increased compared with those at the pod-setting stage. The relative abundance of Pseudomonadota in the F4 treatment was the highest, 23.47%, which was not significantly different from that in the F3 treatment. Only the relative abundance of the F4 treatment was significantly higher than those in the F1 and F2 treatments, 2.80% and 2.21% higher, respectively. Compared with the pod-setting stage, Acidobacteriota in the F1 and F3 treatments decreased slightly, and the relative abundance ratios in the F2 and F4 treatments remained unchanged, but the relative abundance ratio in the F3 treatment was still the highest, and there was no significant difference with the F1 treatment. The relative abundance ratios in the F1 and F3 treatments were still significantly higher than those in the F2 and F4 treatments at the pod-filling stage: 2.13%, 1.42% and 2.90%, 2.19% higher, respectively. There was no significant difference in the relative abundances of Bacillota among the treatments, and F2 was still the highest at 18.04%. The relative abundance of Actinomycetota in the F1 treatment was the highest, accounting for 10.6%, which was significantly higher than those in the other treatments: 1.24%, 2.16% and 1.65%, respectively. Compared with the pod-setting stage, only the F1 and F4 treatments showed an upward trend, while the F2 and F3 treatments decreased.

As shown in Figure 6a, different fertilization strategies can significantly affect the relative abundances of soil bacterial communities at the pod-setting stage. The significance among treatments is shown in Figure S2a. Compared with the F2 and F4 treatments, the F1 and F3 treatments without basal fertilizer significantly increased the relative abundances of *Norank_f_vicinamibacteraceae*, *Norank_o_vicinamibacterales* and *Steroidobacter*; compared with F3, F2 and F4 significantly increased the relative abundances of *Nitrosipira*, *Bacillus*, and *Norank_f_gemmatimonadaceae*. The relative abundance of *Neobacillus* in F2 and F4 was higher than that in F3, but there was no significant difference; the relative abundances of *Nitrospira*, *Bacillus* and *Neobacillus* increased in the F2-F4 treatments compared with those in F1 without fertilization. The F1-F3 treatments significantly increased the relative abundance of *Sphingomonas* compared with the F4 treatment.

As shown in Figure 6b, compared with F2 and F4, the F1 and F3 treatments significantly increased the relative abundances of *Norank_o_vicinaminibacterales* and *Norank_f_vicinaminibacteraceae* and showed an increasing trend compared with the F1 and F3 treatments at the pod-setting stage. The significance among treatments is shown in Figure S2b. There was no significant difference among the *Nitrosipira*, bacillus and *Neobacillus* treatments at the pod-filling stage. Compared with the F1 and F2 treatments, the F3 and F4 treatments at the pod-filling stage significantly increased the relative abundance of *Pseudomonas*. *Sphingomonas* and *Arthrobacter* in the F1 treatment maintained high relative abundance, and only the F1 treatment showed an increasing trend, while the other treatments showed a decreasing trend; the relative abundance of *Sphingomonas* in the F1 treatment was significantly higher than that in the other treatments.

### 2.4. Effects of Fertilization Strategies on Nutrient Uptake and Fertilizer Use Efficiency of Peanut Plants

#### 2.4.1. Nutrient Uptake of N, P and K per Plant in Stems, Leaves and Pods of Peanut

The nutrient uptake of nitrogen (Figure 7a), phosphorus (Figure 7b), and potassium (Figure 7c) by stems, leaves, and pods gradually increases throughout the growth process. For stems and leaves, nutrient uptake reaches its peak during the pod-setting stage and then decreases. During the flowering–pegging stage, F3 exhibited the highest uptake of nitrogen, phosphorus, and potassium. From the pod-setting stage to the maturity stage, F4 consistently exhibited the highest nutrient uptake, with both F3 and F4 significantly surpassing F2. For pods, nutrient uptake steadily increased from the pod-setting stage to the maturity stage. F3 had the highest nutrient uptake during the pod-setting stage, while F4 has the highest nutrient uptake during the pod-filling stage and maturity stages. During the pod-filling and maturity stages, the uptake of nitrogen, phosphorus, and potassium in the pods of F3 and F4 was significantly higher than that of F2. Among them, the nitrogen uptake in the pods of F3 and F4 during the maturity stage was 7.30% and 10.20% higher than that of F2, respectively; the phosphorus uptake of pods was 7.82% and 15.08% higher, respectively; the potassium uptake of pods was 6.99% and 10.05% higher, respectively.

#### 2.4.2. Effects of Fertilization Strategies on Fertilizer Utilization Efficiency of Peanut

The utilization rates of nitrogen, phosphorus and potassium fertilizer in the F4 treatment were the highest, and there was no significant difference with the F3 treatment. The utilization rates of nitrogen, phosphorus and potassium fertilizer in the F3 and F4 treatments were higher than those in the F2 treatment. The utilization rates of N, P and K were 5.98 and 10.78%, 1.54 and 2.31%, and 8.16 and 16.39%, respectively(Table 1).

### 2.5. Mantel Test Correlation Analysis Between Fertilization Strategies and Soil-Available Nutrients, Microbial Community and Peanut Nutrient Uptake and Yield

Mantel test results showed that under different fertilization treatments (Figure 8), AN, AP and AK were positively correlated with environmental factors. There were significant differences in the driving effects of environmental factors on the bacterial community structure. The Chao and Shannon indexes were significantly positively correlated with AN, AP and AK; Pseudomonadota was significantly positively correlated with AP and AK; and Acidobacteriota was negatively correlated with available nutrients. The relative abundances of *Pseudomonas* and *Sphingomonas* were significantly positively correlated with N uptake, P uptake, K uptake and yield.

F1 community was significantly positively correlated with AN, AP, AK and Alp; F2 treatment was significantly positively correlated with AN, AP and Ur; the F3 treatment showed a strong significant positive correlation with AP and AK and a moderate significant positive correlation with AN and Alp; the F4 treatment was only positively correlated with Suc, but not with other environmental factors.

### 2.6. SEM Model Construction and Path Effect Analysis Between Fertilization Strategy and Soil-Available Nutrients, Microbial Community and Peanut Nutrient Uptake and Yield

In this study, the partial least-squares structural equation model (PLS-SEM) was constructed to study the multiple causal relationships among the fertilization strategies, soil-available nutrients, microbial communities and peanut nutrient uptake and yield. The goodness of fit (GOF) of the model is 0.851, indicating that the model has high overall predictive and explanatory power.

The path diagram of the structural equation model (Figure 9a) reveals that the fertilization strategies have a significant positive impact on microbial richness and diversity, key functional bacterial genera, soil-available nutrients, and soil enzyme activity. Soil enzyme activity positively affects soil-available nutrients, reaching a significant level. Soil-available nutrients positively affect both fertilizer use efficiency and nutrient uptake, also reaching a significant level. Nutrient uptake has a significant positive effect on peanut yield and fertility use efficiency. The results indicate that fertilization strategies can directly influence the availability of soil nutrients, thereby promoting nutrient uptake in peanuts and affecting yield formation. Additionally, fertilization strategies can directly affect nutrient availability by regulating soil microbial diversity and structural characteristics, or they can indirectly affect nutrient availability through soil enzyme activity.

The results of the effect decomposition (Figure 9b) indicate that the fertilization strategy primarily exerts an indirect effect on yield, with an indirect effect of 1.30. Both microbial richness and diversity, as well as key functional bacterial genera, exert indirect effects on yield. The total effects of soil enzyme activity and soil-available nutrients on yield are both negative. Nutrient uptake primarily exerts an indirect effect on yield, with an indirect effect of 0.929. The fertilizer utilization rate has the greatest direct effect on yield, with a direct effect of 2.21.

## 3. Discussion

### 3.1. Fertilization Strategy Drives Coordinated Changes in Soil Nutrients and Enzyme Activities in Peanut

The soil-available nutrient content is a direct index for evaluating fertilization effects, and the soil enzyme activity reflects the potential capacity of nutrient transformation. In this study, the contents of alkali-hydrolyzable nitrogen, available phosphorus and available potassium in each treatment peaked at the flowering–pegging stage and then gradually declined, consistent with peanut’s fertilizer demand pattern. The flowering–pegging to pod-setting transition represents the critical stage for both vegetative and reproductive growth, during which nutrient demand is the most vigorous; thus, soil-available nutrients rapidly reach their peak following fertilization [26]. The significantly higher available nutrient contents in F3 and F4 during the late growth stages (pod filling and maturity) align with previous findings demonstrating that drip irrigation with split topdressing sustains soil nutrient availability better than one-time basal application, avoiding late-season nutrient depletion caused by early leaching or fixation [10,27].

Soil enzyme activity is an important biological index to characterize the transformation ability of soil nutrients [28]. In this study, the urease and sucrase activities were consistent with the dynamic trend of soil-available nutrients and gradually decreased after reaching the peak at the flowering–pegging stage. The alkaline phosphatase activity continued to rise from the flowering–pegging stage to the pod-filling stage and reached the peak at the pod-filling stage, which was consistent with the research results of Kumar et al. [29]. Under the condition of drip irrigation and fertilization, Alp reached the peak at 60 days after sowing. In this experiment, there was still drip irrigation and topdressing at the remaining late stage of peanut, and Alp reached the peak at the pod-filling stage and then began to decline. Pang et al. [30] showed that soil enzyme activities (Ur, Suc, Alp) under drip irrigation were significantly higher than those under sprinkler irrigation and furrow irrigation. In this study, the enzyme activity of the F2 treatment was not significantly different from that of F3 and F4 at the flowering–pegging stage, but with the advance of the growth stage, the enzyme activity of the F2 treatment decreased significantly due to the lack of fertilizer supply in the late stage, while the F3 and F4 treatments maintained high soil enzyme activity through fractional drip irrigation and topdressing, which further confirmed the limitation that the one-time application of basal fertilizer does not easily meet the nutrient requirements of peanut throughout the whole growth stage.

An important distinction emerged between the early and late growth stages: at flowering–pegging, enzyme activities did not differ significantly between F2 (one-time basal) and F3/F4 (split applications), but as growth progressed, F2 enzyme activities declined sharply due to late-season nutrient exhaustion, whereas F3 and F4 maintained significantly higher levels. This temporal divergence underscores a fundamental limitation of one-time basal fertilization—it cannot adequately meet the sustained nutrient transformation demands across peanut’s entire growth cycle, particularly during the late reproductive stages when pod development imposes high nutritional requirements. Similar temporal trade-offs have been observed in maize [31] and wheat [32] under drip systems, reinforcing the general principle that synchronizing nutrient supply with crop demand is more critical than total nutrient input.

### 3.2. Regulation of Fertilization Strategies on Stability of Soil Microbial Community Structure

The response of soil bacterial community structures to fertilization strategies is a key indicator for evaluating the ecological effects of fertilization regimes. Long-term fertilization experiments have consistently shown that fertilization-induced changes in soil nutrients significantly alter bacterial community composition, with Pseudomonadota and Acidobacteriota typically dominating farmland soils [20,33]. Our results corroborate these findings, with Pseudomonadota, Acidobacteriota, Bacillota and Actinomycetota constituting the dominant phyla across all treatments. Notably, several of these dominant taxa are recognized as reservoirs of nitrogen-fixing and nodulation-associated bacteria in peanut systems. Pseudomonadota (formerly Proteobacteria), which encompasses *Pseudomonas* and *Bradyrhizobium*, is the primary phylum harboring peanut rhizobia. Within this phylum, *Bradyrhizobium* species are the canonical microsymbionts of peanut, and their presence is essential for effective nodulation and symbiotic nitrogen fixation [4,5]. Although *Bradyrhizobium* was not among the most differentially abundant genera in our differential abundance analysis, its ecological role in peanut nitrogen nutrition warrants consideration when interpreting community-level responses to fertilization. *Pseudomonas*, which was significantly enriched in F4 at the pod-filling stage in our study, includes strains with reported nitrogen-fixing ability and plant growth-promoting traits [34], suggesting that the F4 strategy may favor a functional guild with potential diazotrophic capacity. Similarly, Bacillus—which showed the highest relative abundance in F2 at the pod-setting stage—contains species, such as *B. amyloliquefaciens* and *B. aryabhattai*, that exhibit nitrogen fixation, phosphorus solubilization, and biocontrol activities [34,35]. The comparison of the enrichment patterns of *Pseudomonas* and *Bacillus* between F4 and F2 may reflect the effects of basal fertilization and staged fertilization on these multifunctional groups.

The significantly higher Chao and Shannon indices in F4 compared to F2 (Figure 3) suggest that basal-plus-topdressing drip irrigation enhances bacterial richness and diversity, a finding consistent with Song et al. [24] in summer maize systems. Mantel test correlations further revealed that AN, AP and AK were significantly positively correlated with Chao and Shannon indices, reinforcing the view that higher available nutrient levels support richer microbial diversity [20,36] (Figure 8). SEM path analysis quantified this regulation, with fertilization strategy exhibiting path coefficients of 0.706 for bacterial diversity and 0.725 for key functional genera (Figure 9a), indicating strong regulatory efficacy [37]. NMDS analysis showed that bacterial communities across fertilization treatments had already diverged significantly by the pod-setting stage, with the divergence intensity continuing to increase through pod filling—the ANOSIM R value rose from 0.623 to 0.883 (41.7% increase)—demonstrating that the differentiating effect of fertilization strategies on bacterial communities accumulates progressively with the growth stage (Figure 4).

Importantly, the F3 and F4 treatments exhibited distinct response patterns in terms of community composition and functional characteristics. Pseudomonas was significantly enriched in F3 at the pod-filling stage, where Mantel tests identified available phosphorus and potassium as the primary community drivers. Pseudomonas species are considered typical copiotrophic opportunistic bacteria, capable of rapid proliferation and niche occupation when available nutrients surge suddenly [38]. While such short-term outbreaks of specific genera may promote available nutrient turnover, they may also be associated with a reduction in functional redundancy and, potentially, community stability [39]. This pattern parallels observations in greenhouse vegetable systems, where drip fertigation without organic amendment similarly showed a tendency to favor opportunistic taxa over community evenness [40]. In contrast, multifunctional beneficial genera, such as Paenibacillus, were consistently detected in F4 during the pod-filling stage, and the bacterial community remained decoupled from most environmental factors (except sucrase). The slower decline of Bacteroidota in F4—a phylum reported to play important roles in root exudate utilization and complex polysaccharide degradation [41]—may contribute to the ecological resilience of this treatment. Paenibacillus encompasses multiple plant growth-promoting rhizobacteria with phosphorus-solubilizing and pathogen-antagonistic functions [42]. This decoupled state may be interpreted as a sign of a maturing soil microecosystem with a potentially enhanced buffering capacity and greater functional redundancy [43]. Zhao et al. [44] similarly demonstrated, in a drip-irrigated continuous cropping system, that a stable soil microenvironment with low environmental fluctuation is associated with core microbial network stability and a reduced likelihood of community overresponses to external nutrient disturbances. Moreover, recent meta-analyses have confirmed that fertilization strategies promoting community decoupling—rather than tight nutrient–microbe coupling—generally confer greater resistance to environmental perturbations [45]. Thus, the decoupled state observed in F4 may contribute to a yield and fertilizer use efficiency comparable to F3 and could potentially be associated with the greater long-term ecological resilience of the soil bacterial community at the microecological level [46]—a trait that is widely considered crucial for sustaining farmland ecosystem health.

### 3.3. Synergism of “Time and Space Synchronization” of Fertilization Strategy on Nutrient Uptake and Utilization of Peanut

The observed nutrient uptake patterns at different growth stages show an S-shaped trend, with the peak demand stage from pod formation to pod formation [12], consistent with the general growth rhythm of peanuts. Jain et al. [9] reported that the uptake and yield of nitrogen, phosphorus, and potassium under drip irrigation fertilization were significantly higher than those under conventional fertilization. In our study, F3 and F4 showed significantly higher uptake of nitrogen, phosphorus, and potassium during the pod-setting and pod-filling stages compared to F2 (Figure 7), confirming that precise fertilization during the critical demand stage effectively promoted the accumulation of pod-setting nutrients. These findings are highly consistent with the research results of Chang et al. [11] and Jain et al. [13], who also demonstrated that drip irrigation can improve peanut uptake of nitrogen, phosphorus, and potassium, as well as fertilizer agronomic efficiency. In our accompanying study, the pod yields of F1, F2, F3, and F4 reached 4928.30, 5715.37, 6327.55, and 6257.29 kg · hm^−2^, respectively (Figure S3). F3 and F4 increased by 10.71% and 9.48%, respectively, compared to F2 [47]. These yield increases are comparable to the reported yield increases in drip irrigation systems in the northwest region [7], slightly higher than the average growth observed in traditional drip irrigation studies, which may be due to the precise alignment of the fertilization timing with peak demand windows.

The NPK fertilizer use efficiencies of F4 (N: 46.99%; P: 12.27%; K: 58.39%) were the highest among all treatments, with no significant difference from F3 but significantly higher than F2 (Table 1). This hierarchy mirrors the trends in soil nutrient dynamics (Figure 1) and enzyme activity (Figure 2). In F2, one-time basal application resulted in available nutrients and enzyme activities peaking at flowering–pegging but declining sharply after pod setting due to late-season nutrient depletion, thereby reducing nutrient transformation and uptake efficiency. Moreover, the significantly lower nitrogen use efficiency in F2 (36.21%) may also be partially attributed to the inhibitory effect of excessive basal nitrogen on nodulation and symbiotic nitrogen fixation during the early growth stage [2,3]. When a large amount of basal nitrogen is applied at sowing, peanut plants tend to rely on mineral nitrogen uptake rather than biological nitrogen fixation, which not only reduces the relative contribution of symbiotically fixed nitrogen but may also suppress the development and activity of rhizobial populations in the rhizosphere [3,48]. In contrast, split application through drip fertigation (F3 and F4) delivers nitrogen in smaller demand-matched pulses, which may alleviate the suppression of nodulation and maintain a more balanced contribution from both mineral nitrogen uptake and biological nitrogen fixation [3]. The slightly higher efficiencies in F4 over F3 may be attributed to the basal-plus-topdressing regime more effectively matching peanut’s whole-season nutrient demand, a finding similar to Tao et al. [27], who reported optimal yields at a 40% basal + 60% topdressing ratio in sandy soils under film drip irrigation.

The phosphorus use efficiency of F2 in our experiment was only 9.96%, falling below the typical range of 10–25% reported for peanut under general production conditions [49,50]. This low value is consistent with the “diminishing returns” phenomenon described by Yu et al. [51], who found that when P application exceeds ~90 kg·hm^−2^, P use efficiency and agronomic efficiency begin to decline. Our P application rate (123 kg·hm^−2^) exceeded this threshold, likely contributing to the low P efficiency in F2. However, F3 and F4 maintained normal P efficiency levels despite the same total P input, indicating that split drip application more precisely matches peanut’s stage-specific P demand, reduces P fixation and loss, and improves overall P utilization. This pattern echoes findings in soybean [52], where split P application consistently outperformed single basal applications in high-P-fixing soils.

PLS-SEM path analysis quantified the causal relationships among the variables [53,54] (Figure 9). Fertilization strategy exerted significant positive effects on bacterial community diversity and key functional genera, indicating that adjusting the basal-to-topdressing ratio effectively regulates the richness and functional group composition of soil microbial communities. The strongest positive effect of key functional bacteria on soil enzyme activity suggests that specific biological groups participate in organic matter decomposition and nutrient mineralization by secreting extracellular enzymes, serving as the core intermediary linking fertilization measures to nutrient transformation efficiency [55,56,57]. The effect decomposition results reveal that fertilization strategy’s impact on yield is primarily indirect, operating through the cascading regulation of soil microbial communities, enzyme activities, and nutrient uptake rather than through direct effects (Figure 9b). This supports the core mechanism that fertilization strategy indirectly drives fertilizer efficiency improvement via the “microbial-enzyme-nutrient uptake” cascade. Similar indirect pathways have been identified in rice [58] and wheat–maize rotations [59], where microbial community regulation mediated the majority of fertilization effects on crop productivity. As a multivariate method suitable for complex ecosystems, PLS-SEM can simultaneously address direct and indirect effects among multiple latent variables. Yan et al. [54] used PLS-SEM to explore the driving relationships between soil nutrients, bacterial functional potential, and plant-beneficial species, confirming its applicability in quantifying complex ecological causal chains. In terms of functional bacteria, the relative abundances of Pseudomonas and Sphingomonas were significantly positively correlated with plant NPK uptake and yield [60] (Figure 8), consistent with the SEM-derived inference that “key functional bacteria exert positive effects on nutrient uptake through indirect pathways.” Notably, the negative total effects of soil enzyme activity and available nutrients on yield (Figure 9b) suggest that these factors may have exceeded optimal thresholds in some treatments, or that their direct positive effects were offset by indirect negative feedbacks—a phenomenon also observed in long-term fertilization studies where excessive nutrient availability suppressed beneficial microbial interactions [61].

In addition, although this study did not explore the composition of nodule and rhizobium communities at a deeper level, considering the high nitrogen fertilizer application rate used in this experiment, the potential impact of fertilization strategies on peanut rhizobium symbiosis is worth investigating in future research. It should also be noted that this study is based solely on field results from a single growing season, without accounting for interannual climate variability or differences in initial soil conditions, so the stability and generalizability of the F4 strategy across broader climatic and edaphic contexts require further validation through multi-year, multi-site field trials. Future work should additionally integrate metatranscriptomic or metaproteomic approaches to functionally validate the actual contributions of key microbial communities to nutrient transformation processes.

## 4. Materials and Methods

### 4.1. Test Location and Materials

The experiment was conducted in Baihe Village (114°25′ E, 34°35′ N), Xinghuaying street, Longting District, Kaifeng City, Henan Province, from May to October 2025. The soil type of the test field is sandy loam, and the basic physical and chemical properties of the 0–20 cm soil at the test site are as follows: the organic matter content is 15.40 g · kg^−1^, the alkali-hydrolyzable nitrogen content is 70.13 mg · kg^−1^, the available phosphorus content is 85.17 mg · kg^−1^, and the available potassium content is 161.00 mg · kg^−1^. Yuhua 37 was bred by Henan Academy of Agricultural Sciences and is characterized by a well-developed root system and abundant nodulation, with a relatively high nodulation capacity compared to other peanut cultivars. This variety has been reported as nitrogen-efficient, showing strong symbiotic nitrogen fixation potential when rhizobial populations are properly established. The tested basal fertilizer was compound fertilizer (N: P_2_O_5_: K_2_O-22:10:8), and the water-soluble fertilizer was urea (containing n-46%), monoammonium phosphate (containing N-12%, P_2_O_5_-61%), and potassium sulfate (containing K_2_O-50%). The first crop was garlic.

### 4.2. Experimental Design

The experiment comprised four treatments—F1 (no fertilization), F2 (full basal fertilizer), F3 (full water-soluble fertilizer), and F4 (50% basal fertilizer + 50% water-soluble fertilizer)—each replicated three times in a randomized block design with mechanized ridge planting. Each plot consisted of six ridges (5 m long × 0.8 m wide), with two rows per ridge, a drip irrigation belt laid between the two rows, double-seed sowing, and a planting density of 12,000 holes·mu^−1^; sowing occurred on 28 May 2025, and harvesting on 9 September 2025. The total N-P_2_O_5_-K_2_O inputs were held constant across all fertilized treatments (F2–F4) at 343.9, 123.0, and 120.0 kg·hm^−2^, respectively. Basal fertilizer (22-10-8 compound fertilizer) was applied before ridging and sowing for F2 and F4 (the latter supplying 50% of the total), while F3 received no basal fertilizer. Water-soluble fertilizers used for topdressing—urea (46% N), monoammonium phosphate (12% N, 61% P_2_O_5_), and potassium sulfate (50% K_2_O)—were formulated to achieve the target N-P_2_O_5_-K_2_O ratios for each event. For F3, the water-soluble fertilizer was applied via drip irrigation at a mass ratio of 6:2:2 across the flowering–pegging, pod-setting, and pod-filling stages; for F4, the same topdressing schedule was followed but with a mass ratio of 2:4:4. Both treatments used a Venturi fertilizer applicator for drip fertilization, preceded by 10 min of clean-water irrigation and followed by 30 min of clean-water flushing; the same irrigation timing and duration were applied to F1 and F2. Detailed fertilization amounts for each treatment and stage are presented in Table 2.

The drip irrigation system adopts embedded patch drip irrigation tape, with a drip head spacing of 30 cm (102 drip heads per community), a working pressure of 0.1 MPa, and a drip head flow rate of 2.0 L·h^−1^, and the total amount of irrigation for each treatment is consistent. According to the water demand pattern and rainfall conditions during the growth stage of peanuts, a total of 7 watering sessions were carried out: 1 during the seedling stage (before emergence after sowing), 2 during the flowering stage, 2 during the pod-setting stage, and 2 during the grain-filling stage. The watering amount in the community was evenly distributed each time, with a single watering amount of 51 m^3^ · hm^−2^. The watering time was about 6 h each time, using high-frequency and small-scale watering methods to maintain the soil moisture content in the root zone at 60–80% of the field capacity. The watering time and frequency for handling F1–F4 were completely consistent, except for the difference in whether to fertilize with water or not.

### 4.3. Soil Sample Collection

Five point sampling methods were used to collect 0–20 cm soil layers at the flowering–pegging stage (FPS), pod-setting stage (PSS), pod-filling stage (PFS) and maturity stage (MS). After the roots and impurities were removed by mixing the soil layers of each treatment, one part was dried and sieved to determine the chemical properties of the soil, one part was stored in a 4 °C refrigerator to determine the soil enzyme activity, and the other part was stored in a −80 °C refrigerator to determine the soil microorganisms.

### 4.4. Determination of Soil Nutrients and Enzyme Activities

Soil pH, organic matter, total nitrogen, total phosphorus, total potassium, alkali-hydrolyzable nitrogen, available phosphorus, available potassium and other chemical properties were determined with reference to soil agrochemical analysis methods [62]. Soil enzyme activities were assessed using specific kits, which were purchased from Keming Biotechnology Co., Ltd., Suzhou, China. To measure urease (Ur) activity, 5 g soil was mixed with 1 mL methylbenzene, 10 mL 10% urea solution, and 20 mL citrate buffer (pH6.8) and incubated at 37 °C for 24 h. The mixture was centrifuged at 2000 rpm for 5 min. One milliliter of supernatant was mixed with 4 mL 1.35 mol·L^−1^ sodiumphenolate and 3 mL 1% sodiumhypochlorite. After 20 min, the absorbance of the solution to 578 nm wavelength light was detected. To measure sucrase (Suc) activity, 5 g soil was mixed with 0.25 mL methylbenzene, 5 mL. 8% sucrose solution, and 5 mL phosphate buffer solution (pH 5.5). After incubation at 37 °C for 24 h, the mixture was centrifuged at 2000 rpm for 5 min. An amount of 1 mL supernatant was mixed with 3 mL 0.5% 3,5-dinitrosalicylic acid solution and was heated in a boiling water bath for 5 min. The absorbance of the solution to 508 nm wavelength light was detected. Alkaline phosphatase (Alp) activity was determined using a colorimetric method based on the hydrolysis of disodium phenyl phosphate. The absorbance of the resulting phenol derivative was measured at 578 nm using a multifunction microplate reader (Thermo Scientific Company of the United States, Waltham, MA USA).

### 4.5. Extraction, Amplification and High-Throughput Sequencing of Soil Microbial DNA

According to e.z.n.a ^®^, the specification of the soil DNA Kit (omega bio TEK, Norcross, GA, USA) was used to extract the total genomic DNA of the microbial community, the quality of the extracted genomic DNA was detected by 1% agarose gel electrophoresis, and the concentration and purity of DNA were determined by nanodrop2000 (Thermo Scientific Company of the United States, Waltham, MA, USA). Using the extracted DNA as a template, the v3-v4 variable region of the 16S rRNA gene was amplified by PCR using the upstream primer 338f (5′-actcctacggagggaggcagcag-3′) and the downstream primer 806r (5′-ggactachvggtctaat-3′). The PCR reaction system was: 5 × transstart fastpfu buffer 4 μL, 2.5 mm dNTPs 2 μL, upstream primer (5 μm) 0.8 μL, downstream primer (5 μm) 0.8 μL, and transstart fastpfu DNA polymerase 0.4 μL, 10 ng of template DNA, up to 20 μL. The amplification procedure is as follows: predenaturation at 95 °C for 3 min, 27 cycles (denaturation at 95 °C for 30 s, annealing at 55 °C for 30 s, extension at 72 °C for 30 s), then stable extension at 72 °C for 10 min, and finally preservation at 4 °C (PCR instrument: ABI geneamp ^®^ 9700, Carlsbad, CA, USA). PCR products were recovered by 2% agarose gel, purified by a DNA gel recovery and Purification Kit (PCR clean up kit, Yuhua, China) and detected and quantified by qubit 4.0 (Thermo Fisher Scientific, Waltham, MA, USA). The next flex rapid DNA SEQ kit was used to build a library of purified PCR products: (1) connector linking; (2) magnetic beads were used to screen and remove the self-connected segment of the joint; (3) the library templates were enriched by PCR amplification; (4) the PCR products were recovered by magnetic beads to obtain the final library. Sequencing was performed using the Illumina nextseq2000 platform (Shanghai Meiji Biomedical Technology Co., Ltd., Shanghai, China).

Using fastp (https://github.com/OpenGene/fastp, accessed on 1 August 2026, v. 0.23.4) (HaploX Biotechnology, Shenzhen, China): The software was used to control the quality of the two-terminal original sequencing sequence, and flash was used (http://www.cbcb.umd.edu/software/flash, accessed on 1 August 2026, v. 1.2.11). For software splicing (accessed on 25 August 2026): (1) bases with a mass value of less than 20 at the end of the reads were filtered, and a 10 bp window was set; if the average mass value in the window was less than 20, the back-end bases from the window were cut off, the reads with a mass value of less than 50 bp after quality control were filtered, and the reads containing N bases were removed; (2) according to the overlap relationship between PE reads, the paired reads were merged into a sequence with a minimum overlap length of 10 bp; (3) the maximum mismatch ratio allowed in the overlap region of the spliced sequence was 0.2, and the non-conforming sequences were screened; (4) the samples were distinguished according to the barcode and primers at both ends of the sequence, and the sequence direction was adjusted. The allowable mismatch number of barcodes was 0, and the maximum primer mismatch number was 2. Using uparse V11 software (Drive5 Company) (http://drive5.com/uparse/ accessed on 1 August 2026): According to 97% similarity, the quality control stitched sequences were clustered by operational taxonomic unit (OTU), and the chimera was removed. In order to minimize the impact of the sequencing depth on the subsequent alpha diversity and beta diversity data analysis, the number of sequences of all samples was flattened to 20,000 (recommended). After flattening, the average good’s coverage of each sample could still reach 99.09%. Using the RDP classifier (https://john-quensen.com/tutorials/tutorial-1/, accessed on 1 August 2026, Version 2.11) (Ribosomal Database Project, RDP, East Lansing, MI, USA) compared with the Silva 16S rRNA gene database (v138.2) for OTU species taxonomic annotation, the confidence threshold was 70%, and the community composition of each sample was counted under different species classification levels. picrust2 (v.2.2.0-b) software was used for 16S function prediction analysis.

### 4.6. Determination of Plant Nutrient Content

After cleaning the attached dust with clean water, the plant samples collected in each stage were divided according to the stems, leaves, pods and other organs. They were killed at 105 °C for 30 min, dried at 75 °C to a constant weight, and weighed, and the dry weight of each organ was recorded. After the dried sample was crushed through a 0.5 mm sieve, it was digested by the HNO_3_-HClO_4_-hf combined digestion method. The specific steps are as follows: weigh 0.5 g of crushed sample into a PTFE digestion tank, add HNO_3_ 5 mL, HClO_4_ 2 mL and HF 1 mL, place it on an electric heating plate, digest it at 180 °C until the solution is clear and transparent, remove the acid, fix the volume to 50 mL, and filter it for testing. The total nitrogen content of plants was determined by the Kjeldahl method (H_2_SO_4_-H_2_O_2_ digestion, distillation and titration with Kjeldahl nitrogen determinator); the contents of total phosphorus and total potassium were determined simultaneously by inductively coupled plasma atomic emission spectrometry (ICP-OES) at P 213.618 nm and K 766.490 nm, respectively. The nutrient content of each organ was determined three times, and the quality was controlled by a reference material (gbw10012).

### 4.7. Calculation Formula

Nutrient uptake (mg · plant^−1^) = ∑ (dry matter mass of each organ (g) × nutrient content of each organ (g · kg^−1^)) (1)

Fertilizer utilization rate (%) = (nutrient uptake of peanut in fertilization treatment − nutrient uptake of peanut in nonfertilization treatment)/fertilizer amount × 100%.(2)

### 4.8. Data Analysis

DPS (data processing system) V 7.05 was used to analyze the test data by one-way ANOVA. The least significant difference (LSD) method was used for multiple comparisons of the differences between treatments, and the significance level was set as *p* < 0.05. Excel 2024 software, origin 2024 software and the Meiji bio cloud platform were used to sort out and map the data. In this study, the “plspm” package in R (v.3.6.1) was used in the partial least-squares path analysis (PLS-SEM) to predict the multiple causal relationships between the fertilization strategies, soil properties, microbial communities and peanut nutrient uptake and yield.

## 5. Conclusions

Under equal nutrient input, F4 (50% basal + 50% water-soluble fertilizer via drip irrigation) was the optimal strategy for summer peanut in this experiment. It maintained higher soil nutrient availability and enzyme activity during the late growth stages, improved the N, P and K use efficiency, and increased the yield compared with full basal fertilization. The yield advantage was mainly mediated indirectly through the “microbial community, enzyme activity, nutrient conversion, plant uptake” cascade. These results provide a practical basis for precise drip fertigation management in summer peanut.

## Figures and Tables

**Figure 1:**
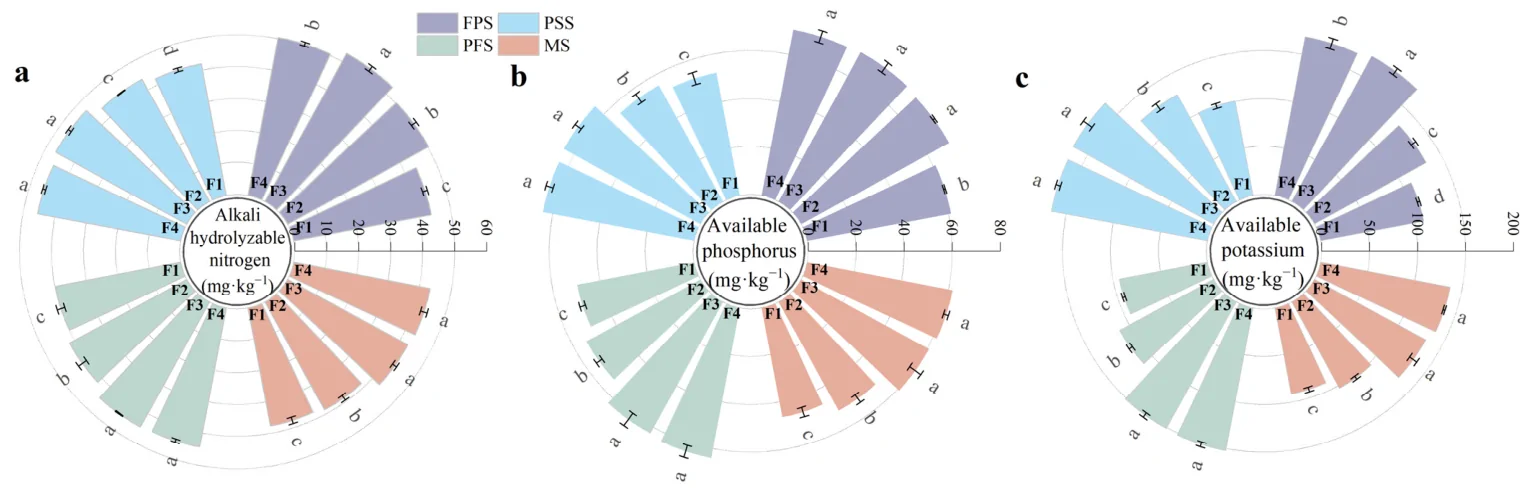
The contents of available nutrients in the 0–20 cm soil layer under different fertilization strategies ((**a**) alkali-hydrolyzable nitrogen (AN); (**b**) available phosphorus (AP); (**c**) available potassium (AK)). The dynamic changes during the four growth stages: the purple bar chart represents the flowering–pegging stage (FPS), the blue bar chart represents the pod-setting stage (PSS), the green bar chart represents the pod-filling stage (PFS), and the orange bar chart represents the maturity stage (MS). Note: The different lowercase letters above the bars indicate significant differences among treatments within the same growth stage and the same soil parameter at *p* < 0.05. Error bars represent standard deviations (*n* = 3).

**Figure 2:**
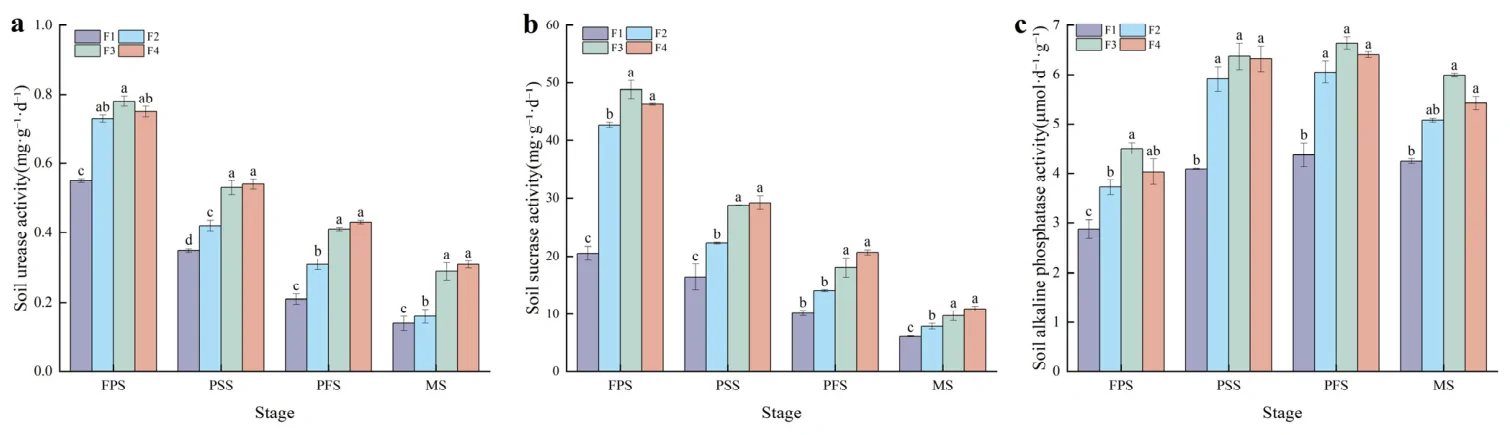
Effects of different fertilization strategies on soil enzyme activities, including (**a**) urease (Ur), (**b**) sucrase (Suc), and (**c**) alkaline phosphatase (Alp), across four growth stages (FPS, PSS, PFS, and MS). Enzyme activities are expressed as mg·g^−1^·d^−1^ for urease and sucrase and as μmol·d^−1^·h^−1^ for alkaline phosphatase. Note: In each growth stage, F1, F2, F3 and F4 were arranged from left to right. Different letters indicate significant differences among treatments in same stage (*p* < 0.05).

**Figure 3:**
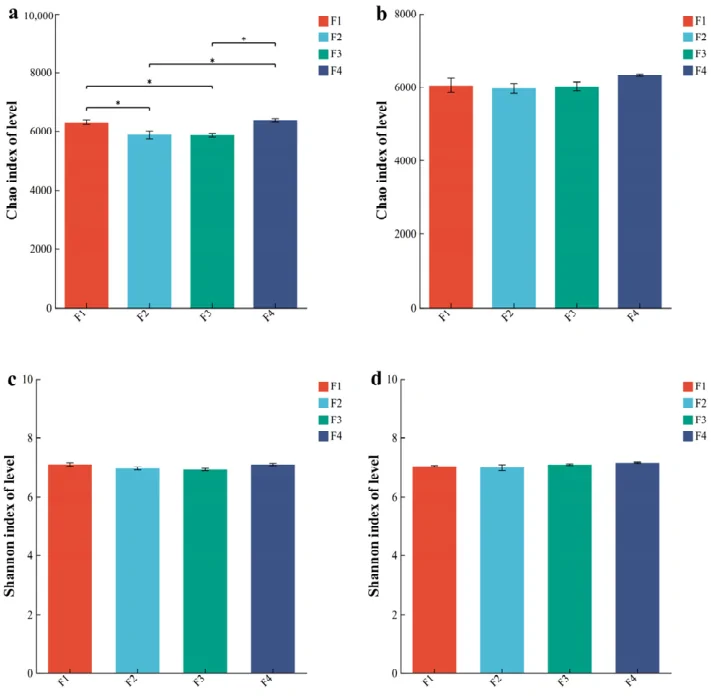
Soil bacterial α diversity (Chao index for richness and Shannon index for diversity) during (**a**,**c**) pod-setting stage (PSS) and (**b**,**d**) pod-filling stage (PFS) under different fertilization strategies. Note: * indicates significant difference between two groups (*p* < 0.05).

**Figure 4:**
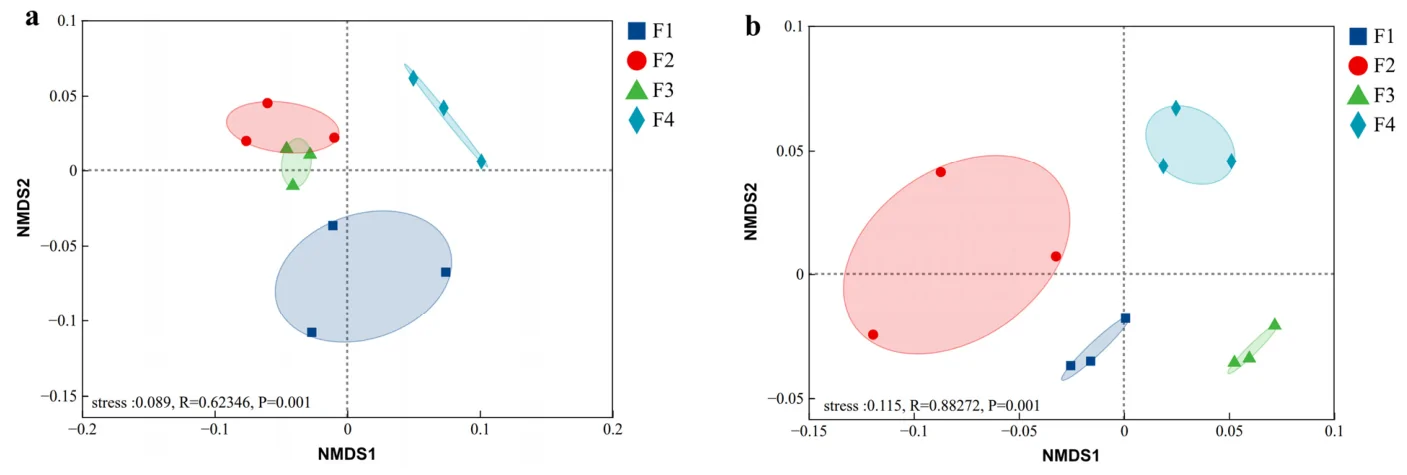
Non-metric multidimensional scaling (NMDS) ordination of soil bacterial communities at OTU level based on Bray–Curtis distance under different fertilization strategies during (**a**) pod-setting stage (PSS) and (**b**) pod-filling stage (PFS). Note: Each point represents individual replicate (*n* = 3). ANOSIM results—PSS: R = 0.623, *p* < 0.001; PFS: R = 0.883, *p* < 0.001—indicating significant separation among treatments. Ellipses represent 95% confidence intervals around group centroids.

**Figure 5:**
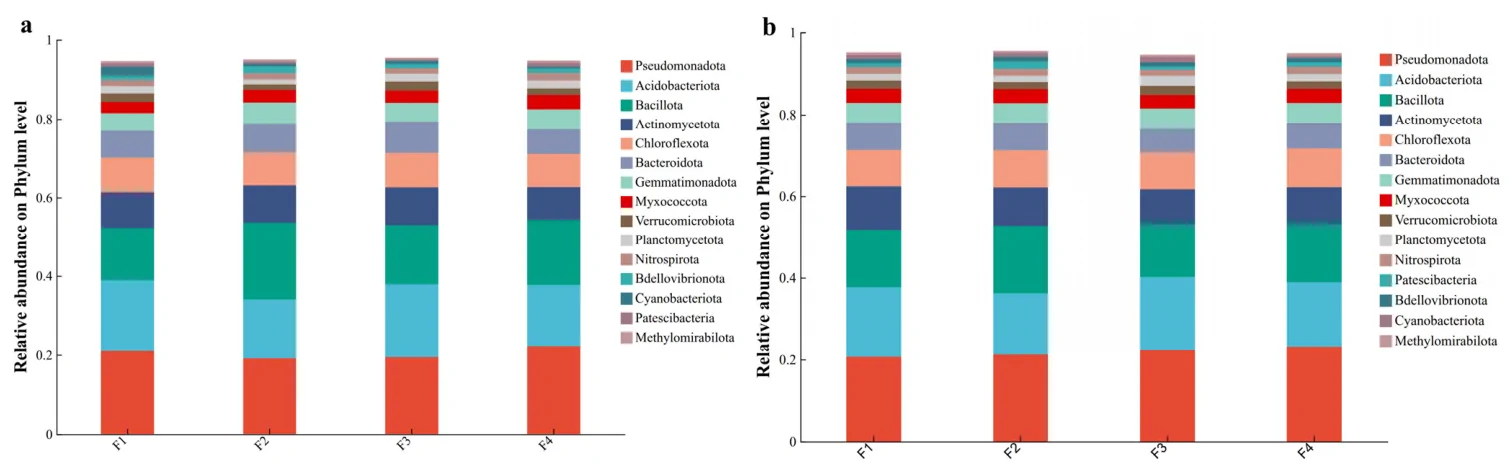
The relative abundances of soil bacterial communities at the phylum level under different fertilization strategies at the (**a**) pod-setting stage (PSS) and (**b**) pod-filling stage (PFS). Note: Only the top 15 phyla in terms of relative abundance among all samples are shown; the remaining phyla are classified as “others”.

**Figure 6:**
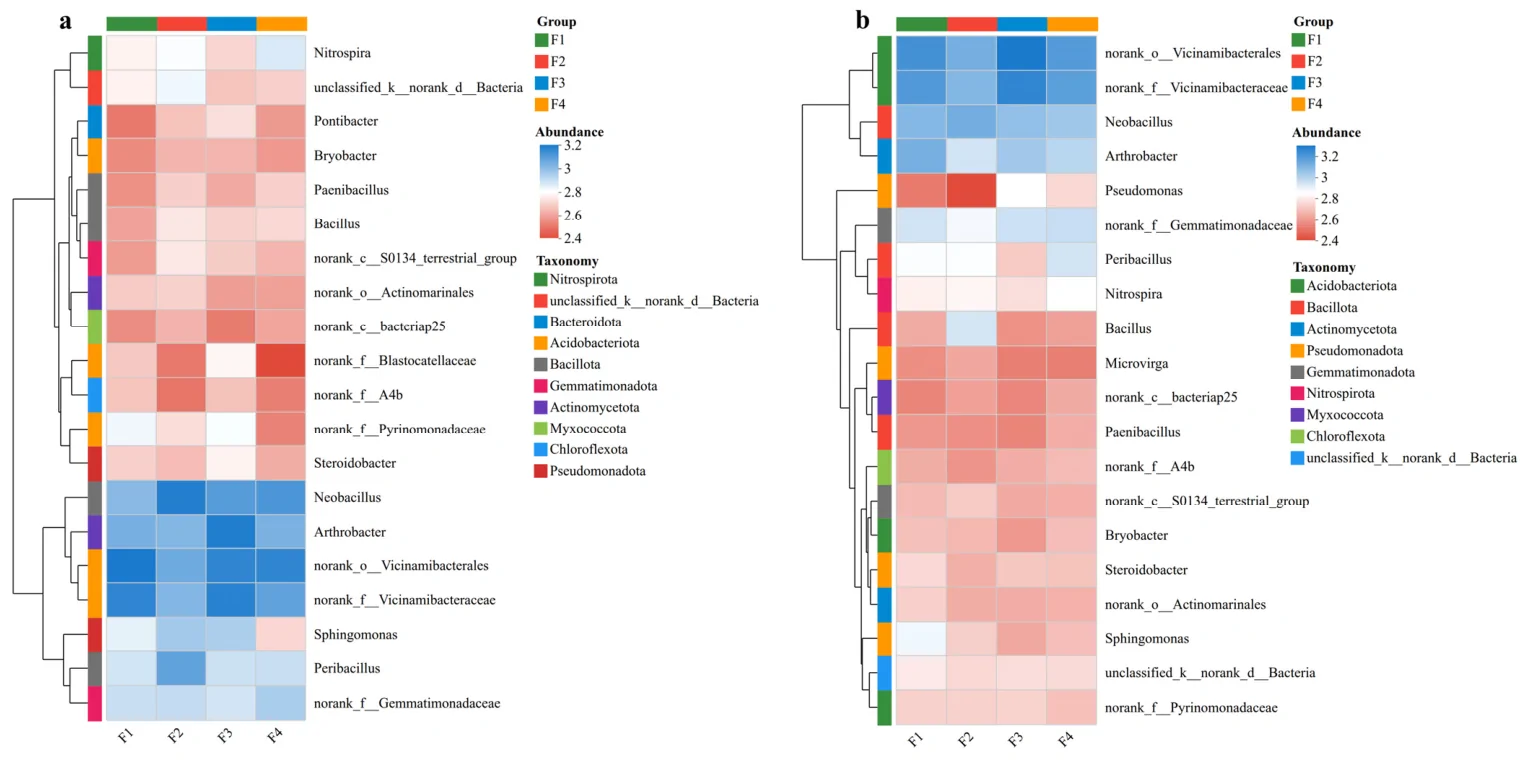
Relative abundances of key differentially abundant bacterial genera under different fertilization strategies at (**a**) pod-setting stage (PSS) and (**b**) pod-filling stage (PFS).

**Figure 7:**
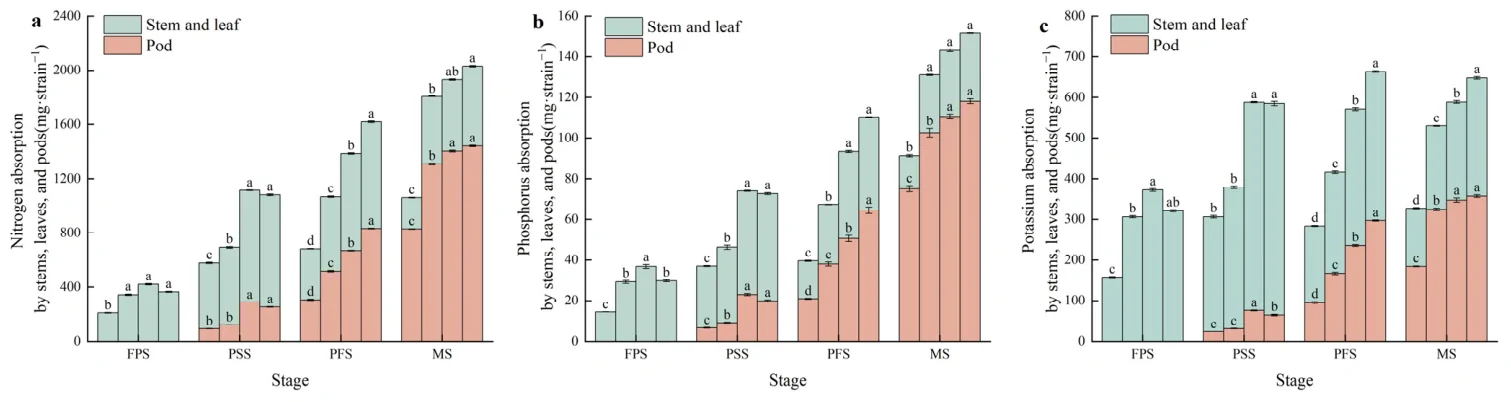
(**a**) Nitrogen (N), (**b**) phosphorus (P), and (**c**) potassium (K) uptake (mg·plant^−1^) in peanut stems + leaves (upper panels) and pods (lower panels) under different fertilization strategies across four growth stages (FPS, PSS, PFS, and MS). Note: In each growth stage, F1, F2, F3 and F4 were arranged from left to right. Different letters indicate significant differences among treatments in the same stage (*p* < 0.05).

**Figure 8:**
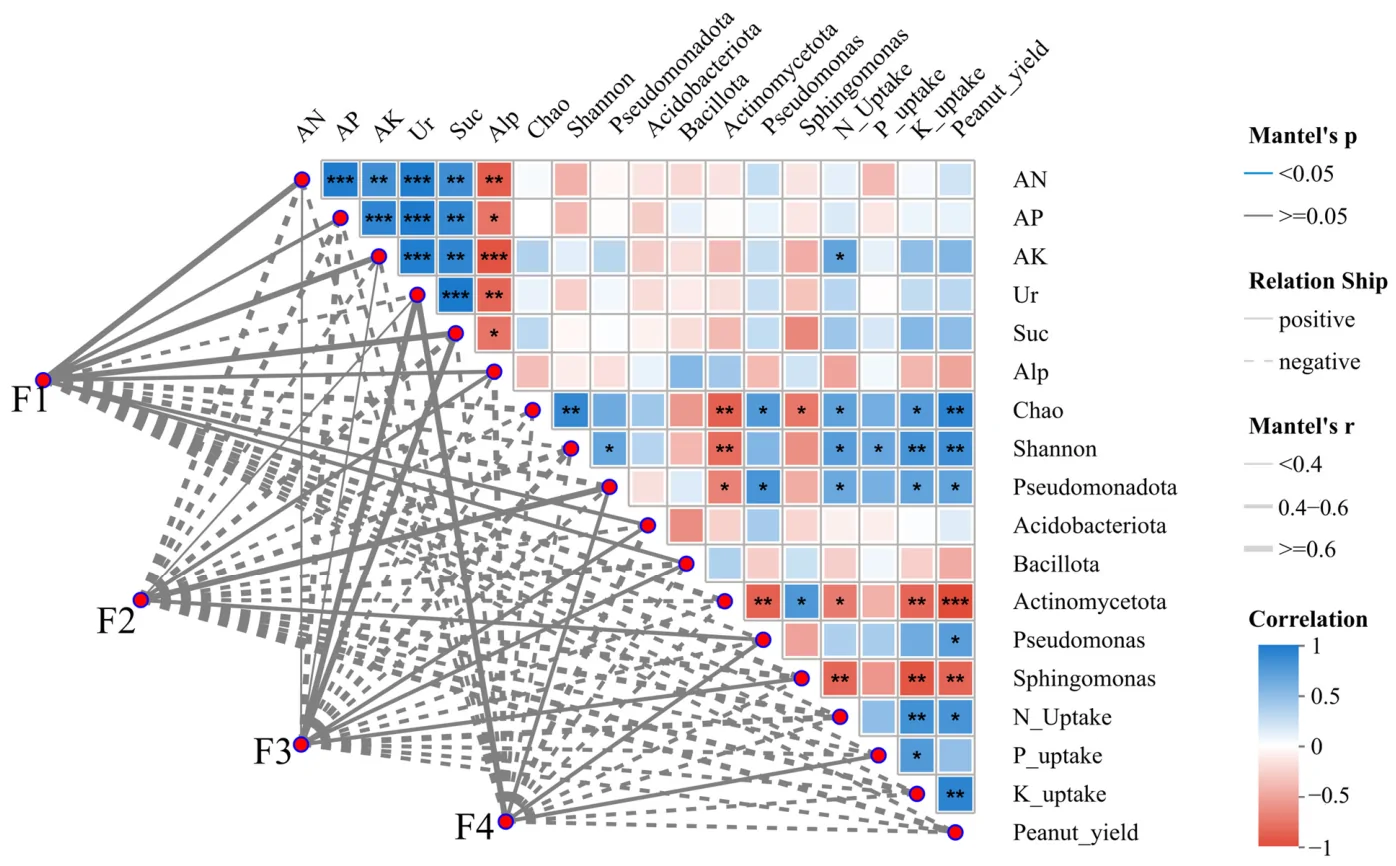
Correlation between soil nutrients, enzyme activity, plant nutrient uptake, and soil microorganisms under different fertilization strategies. Note: Abbreviations: AN, alkali-hydrolyzable nitrogen; AP, available phosphorus; AK, available potassium; Ur, urease; Suc, sucrase; Alp, alkaline phosphatase; N, P, and K uptake, plant nitrogen, phosphorus, and potassium uptake; Chao, Chao richness index; Shannon, Shannon diversity index. Mantel’s r value reflects strength of correlation between environmental factors (available nutrients and enzyme activity) and community distance matrix (r ≥ 0.6 indicates strong correlation; 0.4 ≤ r < 0.6 indicates moderate correlation; and r < 0.4 indicates weak correlation), with *p* < 0.05 indicating statistical significance.The asterisks in the chart represent: * *p* < 0.05, ** *p* < 0.01, *** *p* < 0.001.

**Figure 9:**
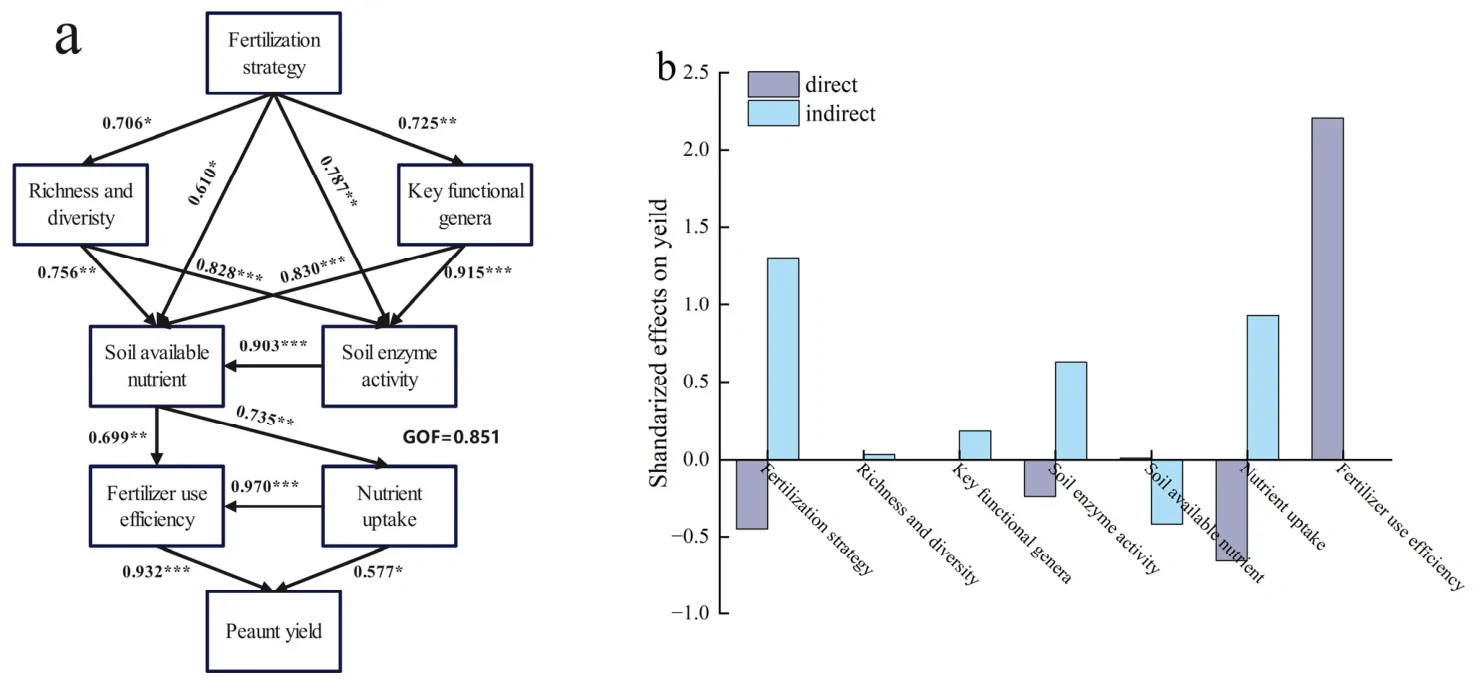
The structural equation model and effect decomposition of the soil properties–bacterial community–plant nutrient uptake–yield under different fertilization strategies. Note: (**a**) The PLS-SEM path analysis diagram. The rectangles represent observation variables and the ellipses represent latent variables. The solid arrow indicates that the path coefficient is significant (* *p* < 0.05, ** *p* < 0.01, *** *p* < 0.001), and the dotted arrow indicates that the path coefficient is not significant (*p* > 0.05). The number on the arrow is the standardized path coefficient. The value of R^2^ indicates the explanatory degree of each endogenous potential variable. (**b**) Decomposition of the direct effect, indirect effect and total effect of each potential variable on the yield.

**Table 1:** Utilization rates of nitrogen, phosphorus, and potassium fertilizers under fertilization strategies (%).

Treatment	Nitrogen Utilization Rate	Phosphorus Utilization Rate	Potassium Utilization Rate
F1	-	-	-
F2	36.21 b	9.96 a	42.00 b
F3	42.19 a	11.50 a	50.16 a
F4	46.99 a	12.27 a	58.39 a

Note: Different lowercase letters indicate significant differences among treatments at *p* < 0.05.

**Table 2:** Fertilization strategy (N-P_2_O_5_-K_2_O) (kg·hm^−2^).

Treatments	Basal Fertilizer	FPS	PSS	PFS
F1	—	—	—	—
F2	343.9-123.0-120.0	—	—	—
F3	—	206.3-73.8-72.0	68.8-24.6-24.0	68.8-24.6-24.0
F4	172.0-61.5-60.0	34.3-12.3-12.0	68.8-24.6-24.0	68.8-24.6-24.0

Note: FPS: flowering–pegging stage; PSS: pod-setting stage; PFS: pod-filling stage. F1: no fertilization; F2: full basal fertilizer; F3: full water-soluble fertilizer applied via drip irrigation; F4: 50% basal fertilizer + 50% water-soluble fertilizer.

## Data Availability

The original contributions presented in this study are included in the article/Supplementary Materials. Further inquiries can be directed to the corresponding author.

## Supplementary Materials

The following supporting information can be downloaded at: https://www.mdpi.com/article/10.3390/plants15192949/s1, Figure S1: Significant differences in soil bacterial phylum levels in top 15 of relative abundance under different fertilization strategies at (Figure a) pod-setting stage (PSS) and (Figure b) pod-filling stage (PFS). Figure S2: Under different fertilization strategies (Figure a), there were significant differences in the relative abundances of the key differential abundance bacteria genera at the pod-setting stage (PSS) and (Figure b) pod-filling stage (PFS). Figure S3 is a supplementary chart for peanut yield.
