# Supplementary material for: Drip Irrigation and Fertilization Strategies Shape Soil Bacterial Community and Nutrient Utilization in Summer Peanut
Source: Plants (Basel). 2026 Sep 27;15(19):2949. doi: 10.3390/plants15192949 (PMC13666724; doi:10.3390/plants15192949)
Supplement: Supplementary file 1 [file plants-15-02949-s001.zip › plants-4564832-supplementary.pdf]

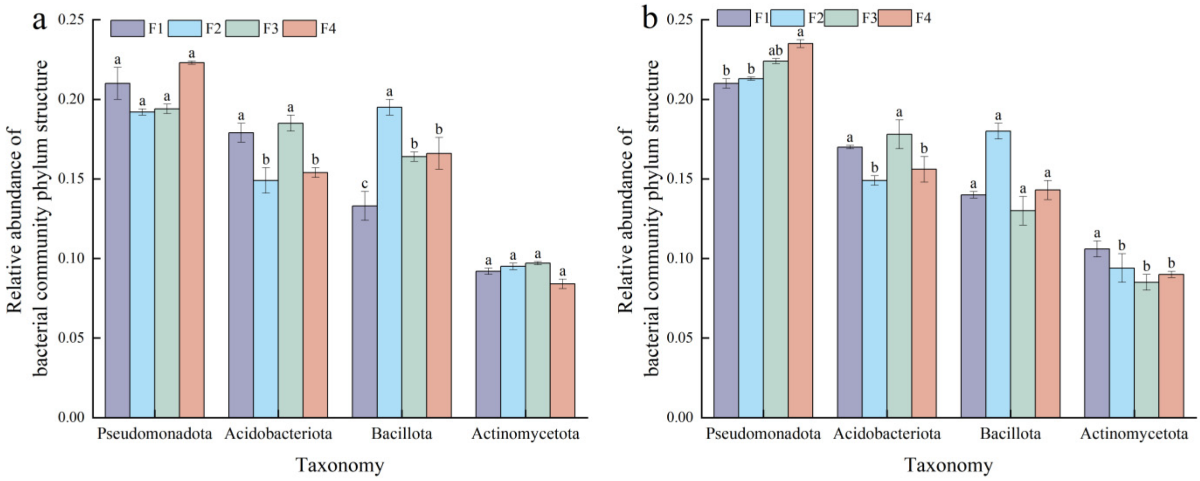

Fig S1. Significant differences of soil bacterial phyla levels in the top 15 of relative abundance under different fertilization strategies at (a) pod setting stage (PSS) and (b) pod filling stage (PFS)

Note: In each growth stage, F1, F2, F3 and F4 were arranged from left to right. Different letters indicate significant differences among treatments in the same stage (*P*<0.05).

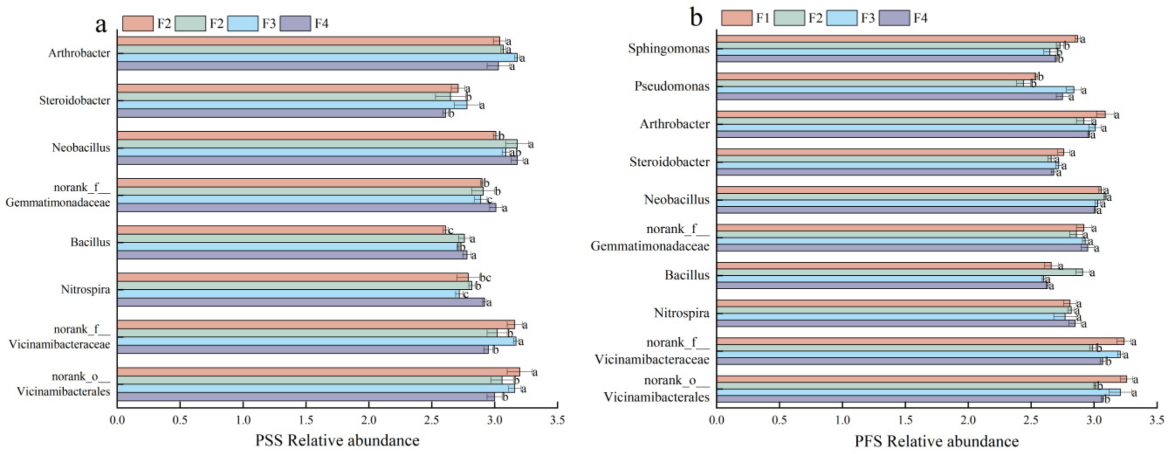

Fig S2. Under different fertilization strategies (a), there were significant differences in the relative abundance of the key differential abundance bacteria genera at the pod setting stage (PSS) and (b) at the pod filling stage (PFS).

Note: At each growth stage, F1, F2, F3 and F4 were arranged from top to bottom. Different letters indicate significant differences among treatments at the same stage (*P*<0.05)

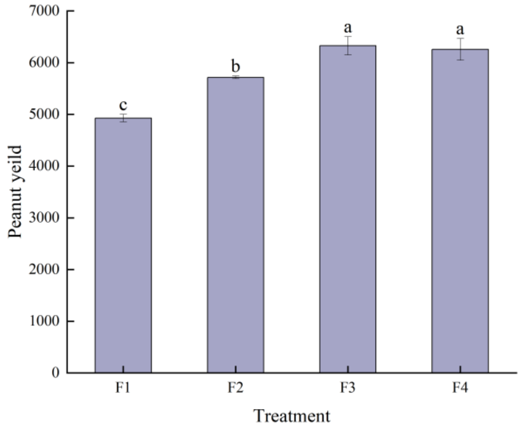

Fig S3. The effect of fertilization strategies on yield

Note: Different letters indicate significant differences between treatments (P<0.05)
